# Evaluation of Potential for Nature-Based Recreation in the Qinghai-Tibet Plateau: A Spatial-Temporal Perspective

**DOI:** 10.3390/ijerph19095753

**Published:** 2022-05-09

**Authors:** Yayan Lu, Fang Han, Qun Liu, Zhaoguo Wang, Tian Wang, Zhaoping Yang

**Affiliations:** 1 State Key Laboratory of Desert and Oasis Ecology, Xinjiang Institute of Ecology and Geography, Chinese Academy of Sciences, Urumqi 830011, China; luyayan16@mails.ucas.ac.cn (Y.L.); hanfang@ms.xjb.ac.cn (F.H.); wangtian181@mails.ucas.ac.cn (T.W.); 2 University of Chinese Academy of Sciences, Beijing 100049, China; 3 Fujian Provincial Key Laboratory for Subtropical Resources and Environment, Fujian Normal University, Fuzhou 350007, China; liuqun@fjnu.edu.cn; 4 College of Economic and Management, Shenyang Agricultural University, Shenyang 110866, China; wzg@syau.edu.cn

**Keywords:** nature-based recreation, mountain landscapes, spatial-temporal dynamics, sustainable management, Qinghai-Tibet plateau

## Abstract

Nature-based recreation (NBR) is an important cultural ecosystem service providing human well-being from natural environments. As the most concentrated and high-quality wilderness in China, the Qinghai-Tibet Plateau (QTP) has unique advantages for NBR. In this study, we designed an integrated nature-based recreation potential index (INRPI) based on four aspects: nature-based recreation resources, landscape attractiveness, recreation comfort and opportunity, and recreation reception ability. A combination of the analytic hierarchy process (AHP) and entropy evaluation method was adopted to assess the NBR potential in the QTP from 2000 to 2020. The research shows that: (i) The INRPI for the QTP decreases gradually from southeast to northwest and increases slightly from 2000 to 2020. (ii) The INRPI displays a pronounced difference on either side of the Qilian-Gyirong line. The areas with very high and high potentials mainly distributed in the southeast of the line, while areas with very low and low potentials distributed in the northwest. (iii) The construction of protected areas effectively improves NBR potential. Areas of INRPI at diverse levels within protected areas obviously increased in 2020. (iv) Increasing altitude has a notable effect on INRPI, and 3000 m is a critical dividing line for the NBR in the QTP. These findings can contribute to decision-makers in guiding rational use and spatial planning of natural land and promoting sustainable recreational development.

## 1. Introduction

Nature-based recreation (NBR) is an important cultural ecosystem service comprising all physical and intellectual interactions with biota, ecosystems, and land-/seascapes [1]. Nature-based recreation primarily refers to access to and exposure to natural landscapes and environments for experiencing nature [2,3]. There is an array of nature-based activities that can be considered NBR, such as hiking, cycling, climbing, and wildlife photography. The emerging consensus is that NBR is an important ecosystem service that connects nature conservation and community development [4,5]. NBR follows the UN Sustainable Development Goals (SDGs) by effectively reducing threats to ecosystems and natural habitats [2]. Furthermore, NBR also serves as a source of income and livelihood for local communities [2] to alleviate poverty [6]. Therefore, recognizing the recreational value of natural areas can bridge the gap between ecological conservation and sustainable tourism.

The COVID-19 pandemic remarkably reduced the number of tourists [7]. However, there has been an increase in public nature recreation participation [8], which greatly benefits individuals and societies by improving physical, psychological, and emotional well-being [9,10,11]. To address the imbalance between recreational supply and demand in post-epidemic periods, it is essential and meaningful to identify regions suited to NBR development by local governments and managers responding to the pandemic.

Extra-large and high-quality wilderness patches are distributed widely in the Qinghai-Tibet Plateau (QTP) [12]. It is one of the most attractive recreational destinations in China and the world because of its authenticity and splendid scenery [13]. In recent decades, there has been noticeable and continued growth in tourists visiting the QTP for mountaineering, hiking, climbing, wildlife photography, and trekking [14]. According to the statistical yearbooks, tourists traveling to Qinghai and Tibet exploded from 3.82 million to 107.14 million from 2000 to 2020, an increase of nearly 28 times. This emphasis on the full utilization of specific landscapes in the QTP for NBR is an important basis for tourism economic development. In 2021, the Chinese government set up two national parks (Three-River-Source and Giant Panda) and will promote the integration of Mount Qomolangma, Qiangtang, and other regions in the QTP into the spatial layout of the national parks [15]. NBR is predominantly supported and encouraged as a solution to the contradiction between ecological conservation and community development in protected areas. This buttresses the call for the goals and philosophy of Chinese national parks, namely, ecological conservation, national representation, and public welfare.

The evaluation for NBR potential is the foundation for regional recreation planning, investment, and management strategy. Thus, spatial layout and zoning are widely regarded as vital management tools [16]. This method has been used extensively for various tourism purposes, including ecotourism [16,17], glacier tourism [18], and natural-based tourism [3,6]. Although ecotourism is considered as a partially overlapping sub-category of NBR [2], it has long been the focus of research [3]. There is limited available research on NBR potential, except for protected areas [5,19], the national scale [6,11], and the transnational scale [20]. There is a general dearth of corresponding studies in plateau areas. The unique climate, topography, biodiversity, and ecosystems in plateau regions make them distinct from other regions. Therefore, the spatial distribution of regions suitable for NBR and their formation mechanisms may differ. In addition, a substantial proportion of available research assesses only the suitability and potential of NBR for selected periods. This creates a stereotypical impression that there is no change in NBR potential. It is becoming increasingly evident that economic uplift, road construction [21], and climate change [22] are altering the spatial patterns of recreational potential. A particular focus should be on the temporal and spatial dynamic evolution of NBR and relating such changes to future recreation development planning and sustainable management. Thus, in comparison with research efforts found in the literature, the highlight of this research is to address the lack of evaluation of the spatiotemporal dynamics of NBR potential in plateau regions.

Research into the products [23], behaviors [24], and sustainability [25] of NBR spawned concerns about suitable regions in the QTP for NBR. Researchers have long attached immense importance to the single-factor evaluation of NBR, including climate suitability [22,26], health risks of hypoxia [13,27], and transport accessibility [21]. These efforts helped us to identify the factors influencing NBR, though it is difficult to determine the potential of NBR based on a single-factor evaluation. Recently, an assessment framework consisting of the suitability and carrying capacity of NBR was proposed [15], which provides a powerful contribution to understanding natural recreation function and planning. However, the quantitative evaluation and spatial identification of NBR potential assessments have largely been overlooked. Therefore, a spatialized NBR potential based on a multi-index in the QTP is urgently needed.

In this study, we focused on NBR spatiotemporal dynamics in the QTP from 2000 to 2020. First, we developed an evaluation system of the NBR potential suitable for the QTP and assigned a weight to each indicator based on subjective and objective methods. Second, we systematically analyzed the temporal and spatial variations of the integrated nature-based recreation potential index (INRPI) to provide scientific references for NBR sustainable management. Section 2 describes the study area, evaluation system, data, and methods. Section 3 evaluates the spatiotemporal distribution characteristics of the INRPI, focusing on the effects of the Qilian-Gyirong line, protected areas, and terrain gradient. The influencing factors and policy implications of these findings are discussed in Section 4, and Section 5 presents our conclusions.

## 2. Materials and Methods

### 2.1. Study Area

**Figure 1:**
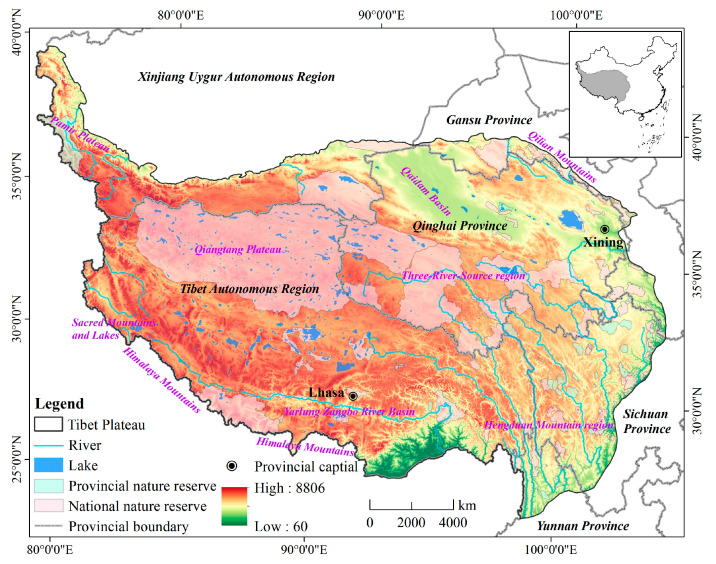
The location of study area (Map number: GS (2020)4619).

The Qinghai-Tibet Plateau, known as the third pole and roof of the world, is located at 25°49′55″–39°49′17″ N and 73°30′4″–104°41′7″ E (Figure 1), with a total area of 262.89 × 10^4^ km^2^. The average elevation is over 4000 m above sea level. The plateau has a typical high-elevation climate, with temperature and precipitation decreasing from southeast to northwest [14]. The long-term mean annual temperature and precipitation are 4.3 °C and 450 mm, respectively [28]. The QTP covers the Tibet Autonomous Region, Qinghai Province, Xinjiang Uygur Autonomous Region, Gansu Province, Sichuan Province, and Yunnan Province.

The QTP is a mysterious and attractive place because of its unique natural and distinctive cultural ecosystems [15]. These recreational resources (e.g., high mountains, glaciers, lakes, and grasslands) make it a world-renowned tourist destination. The number of tourism attractions in Tibet and Qinghai on the list of Chinese national A level scenic spots rapidly increased from 111 in 2010 to 439 in 2020. Over the past 20 years, the number of tourists in Qinghai and Tibet increased by 15 and 65 times, respectively. Tourism income increased by 52 and 82 times, respectively. In addition, in 2020, the proportion of tourism revenue in Qinghai and Tibet in the national economy exceeded 33% and 16%, respectively. Tourism became an important engine for stimulating economic growth.

### 2.2. Research Framework

**Figure 2:**
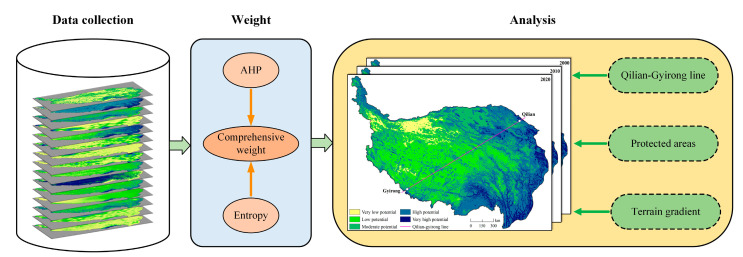
Research framework of this study.

We first build the evaluation system suitable for the QTP and collect the corresponding data. Then, the weight for each indicator is assigned using the AHP and entropy method. Finally, we generate the NBR potential maps in 2000, 2010, and 2020. The line connecting Qilian and Gyirong Counties (Qilian-Gyirong line) is a critical geographical division line in the QTP [29]. The southeast and northwest regions of the Qilian-Gyirong line have almost the same areas. This line can characterize the regional differentiation characteristics of NBR. Protected areas are important supply areas for cultural ecosystem services such as recreation and spiritual enjoyment [25,30]. The complex topography in QTP largely determines the spatial distribution of recreation service [31]. Thus, we analyze the spatial and temporal differences from three aspects: the Qilian-Gyirong line, protected areas, and terrain gradient. The research framework is provided in Figure 2.

### 2.3. Establishment of NBR Potential Index System

The development potential of NBR is related to its resource types, size, attractiveness, spatial structure, location conditions, and socioeconomic support. NBR resources, relief conditions, and vegetation coverage are the natural bases that support NBR function. Their spatial patterns determine the suitability and carrying capacity of NBR utilization [15]. Concurrently, the level of economic development and infrastructure conditions can also enhance the experience for tourists [18]. Focusing on the NBR potential evaluation of the target layer, four criterion layers of nature-based recreation resources, landscape attractiveness, recreation comfort and opportunity, and recreation reception ability were defined. Furthermore, the judging matrices of 15 evaluation indicators within the four criterion layers were designed (Table 1).

**Nature-based recreation resources** are an important first step in identifying priorities for regional NBR development. Generally, tourists would prefer to travel to places with abundant, distinctive, and diverse resources. In this study, the resource value was evaluated from three aspects: landscape diversity (SHDI), landscape heterogeneity (RDLS), and biodiversity (HQ). Landscape diversity refers to the number of landscape elements and the proportion of each landscape element within a certain spatial range [6]. Natural resources such as forests, grasslands, and lakes are the resource basis for NBR, and benefits to tourists increase with increasing resource diversity. Shannon’s diversity index is widely utilized to measure landscape diversity [32]. Landscape heterogeneity refers to the spatial change of landscape elements and their combinations, increasing landscape aesthetic value and improving the recreation experience. A nonuniform land surface with systematic regional differences is a key index that reflects landscape differences [33]. The QTP serves as a global hotspot for biodiversity [34], which has one of the strongest influences on NBR [5].

**Landscape attractiveness** represents the natural potential and capacity of a landscape to support NBR [32,35]. Indicators, including distance to protected areas (DTPA), vegetation coverage (NDVI), distance to lakes (DTL), distance to rivers (DTR), and distance to glaciers (DTG) were chosen to characterize landscape attractiveness. Protected areas are more attractive to NBR because of their outstanding natural landscapes and biodiversity [6,19]. The higher the vegetation coverage, the higher is the landscape attraction. In the QTP, landscape attraction is distributed over large areas of glaciers [26,28] and many plateau lakes [36]. These water resources can create astonishing and exciting environments, attract tourist attention, and provide more recreational opportunities [3,17].

**Recreation comfort and opportunity** are determinants of destination choice. They affect the physical and mental feelings of tourists [37,38]. The indicators of oxygen content (OC), plateau reaction risk index (PRRI), mean annual temperature (TEM), mean annual precipitation (PRE), and terrain niche index (TNI) were selected to characterize the recreation comfort and opportunity. Many tourists dare not travel to plateau areas for fear of health and life safety under high altitudes, such as sleep disturbances, high-altitude headaches, and acute mountain sickness [39]. Climate comfort, such as temperature and precipitation, must be considered for tourists because they can affect travel motivation, destination choice, and travel time [22]. Terrain conditions have a pronounced influence on recreational opportunities. The higher the elevation and steeper the slope, the more difficult it is to approach, and the suitability for recreation decreases.

**Recreation reception ability** is one of the most crucial support systems for the sustainable development of NBR [18]. We chose distance to county (DTC) and traffic accessibility (TA) to describe the regional recreation reception ability. Concurrently, recreation reception facilities (e.g., hotels, restaurants) are mainly concentrated in counties. Traffic accessibility reflects the support capacity of transport facilities and increases tourist willingness to visit a place.

**Table 1:** Calculations of NBR potential index system in the QTP.

Criteria	Indicator and Attribute	Units	Data Source and Computation	Spatial Resolution
Nature-based recreation resources	Landscape diversity (+)	—	Calculated by Fragstats 4.2 software. The land use and land cover data was provided by Huang et al. [40]	30 m
	Landscape heterogeneity (+)	—	Data from the National Tibetan Plateau Data Center (https://data.tpdc.ac.cn/ (accessed on 20 February 2022))	1 km
	Biodiversity (+)	—	Calculated by InVEST based on reference [41]	1 km
Landscape attractiveness	Distance to protected areas (−)	m	Calculated by the “Euclidean distance” tool in ArcGIS	1 km
	Vegetation coverage (+)	—	Data from the MODIS vegetation index product (https://www.usgs.gov/ (accessed on 20 February 2022)), using the maximum value composite (MVC) method to obtain the annual NDVI data	250 m
	Distance to lakes (−)	m	Calculated by the “Euclidean distance” tool in ArcGIS	1 km
	Distance to rivers (−)	m	Calculated by the “Euclidean distance” tool in ArcGIS	1 km
	Distance to glaciers (−)	m	Calculated by the “Euclidean distance” tool in ArcGIS	1 km
Recreation comfort and opportunity	Oxygen content (+)	g/m^3^	Based on the reference [26]	1 km
	Plateau reaction risk index (−)	%	Based on the reference [27]	1 km
	Temperature (+)	°C	Data from the National Tibetan Plateau Data Center (https://data.tpdc.ac.cn/ (accessed on 20 February 2022))	1 km
	Precipitation (+)	mm	Data from the National Tibetan Plateau Data Center (https://data.tpdc.ac.cn/ (accessed on 20 February 2022))	1 km
	Terrain niche index (−)	—	Based on the reference [31]	1 km
Recreation reception ability	Distance to county (−)	m	Calculated by the “Euclidean distance” tool in ArcGIS	
	Transport accessibility (−)	h	Based on the reference [21]	1 km

Note: “+” represents a positive indicator; “−” represents a negative indicator.

### 2.4. Weight Determination Methods

It is essential to assign a weight to each indicator before evaluating the NBR potential. The analytic hierarchy process (AHP) and entropy evaluation method (EEM) were adopted to calculate the weights.

#### 2.4.1. Determining the Subjective Weight Using the Analytic Hierarchy Process

The AHP, proposed by Saaty [42], is a multi-criterion and multi-objective decision-making method that combines qualitative and quantitative analyses. There were four main phases: (1) finding suitable indicators and designing a hierarchical analysis structure model, (2) calculating a pairwise comparison between the evaluation indicators for each level of the hierarchy, (3) calculating the relative weights, and (4) testing the consistency of the judgment matrix by consistency ratio (CR < 0.1) [18,35]. In February 2022, we invited eight experts from different professional backgrounds (physical geography, human geography, landscape ecology, tourism management, ecology, and nature education) to form groups of experts. More than half of these experts have researched or traveled to the QTP. The experts were invited to determine the relative importance of the evaluation indicators. If the consistency ratio was greater than 0.1, the relative priority of the judgment matrix was rescored.

#### 2.4.2. Determining the Objective Weight Using the Information Entropy

Information entropy, which reflects the degree of system disorder, is an objective weighting method [43]. The smaller the entropy value of the indicator, the larger the effective information provided by the indicator, and the greater the weight. Information entropy can be expressed using Equations (1) and (2).

(1) $$E_{j}=-\frac{1}{\ln\left(n\right)}\sum_{i=1}^{n}P_{ij}\ln\left(P_{ij}\right)$$

(2) $$W_{j}^{EEM}=\frac{1-E_{j}}{\sum_{j=1}^{m}\left(1-E_{j}\right)}$$

where, $P_{ij}$ is the feature weight of the indicator; $E_{j}$ is the information entropy; $W_{j}^{EEM}$ is the entropy weight. We stipulate that $E_{j}=0$ when $P_{ij}=0$.

#### 2.4.3. Comprehensive Weight

To derive a comprehensive weight with less uncertainty, the theory of minimum relative information entropy was utilized to obtain the combination weight [44]. It reflects the decision information and expresses expert knowledge and practice experiments. The comprehensive weight (Table 2) of each indicator is calculated as follows:

(3) $$W_{j}=\frac{{\left(W_{j}^{AHP}W_{j}^{EEM}\right)}^{0.5}}{\sum_{j=1}^{15}{\left(W_{j}^{AHP}W_{j}^{EEM}\right)}^{0.5}}$$

In Equation (3), $W_{j}$ is the comprehensive weight; $W_{j}^{AHP}$ and $W_{j}^{EEM}$ are the analytic hierarchy process weight and the entropy weight, respectively.

### 2.5. Integrated Nature-Based Recreation Potential Index

First, all indicators were preprocessed using ArcGIS 10.6, including mosaic, clipping, and reprojection. We then resample these indicators to a 1 × 1 km spatial resolution to achieve good spatial consistency.

Considering that the measurement units and attributes of each indicator are inconsistent, it is essential to standardize the index to perform further comparative and comprehensive analyses. Equations (4) and (5) standardize the positive and negative indicators, respectively.

(4) $$X^{s}=\frac{x_{i}-x_{imin}}{x_{imax}-x_{imin}}$$

(5) $$X^{s}=\frac{x_{imax}-x_{i}}{x_{imax}-x_{imin}}$$

where $X^{s}$ is the standardized value of indicator *i*; $x_{i}$ is the value of indicator *i*; $x_{imin}$ and $x_{imax}$ are the minimum and maximum values of indicator *i*, respectively.

Each indicator expresses the potential of various aspects of NBR. Therefore, it is necessary to access the integrated nature-based recreation potential index. Thus, it was calculated using the weighted integrated method. The grid-weighted overlap function of ArcGIS was utilized to generate the spatialized INRPI for the past two decades. The final INRPI value was obtained using Equation (6):

(6) $$\mathrm{INRPI}=\sum_{i=1}^{15}W_{i}{\times X}_{i}$$

where INRPI is the integrated nature-based recreation potential index; $W_{i}$ is the comprehensive weight of indicator *i*; $X_{i}$ is the standardized value of indicator *i*.

From 2000 to 2020, the value of INRPI ranges from 0.22 to 0.84. To understand the spatial differences and temporal changes better, the INRPI was divided into five levels using the geometrical interval method in ArcGIS 10.6. The recreation potential was categorized in levels of very low potential (0.22~0.31), low potential (0.31~0.37), moderate potential (0.37~0.46), high potential (0.46~0.61), and very high potential (0.61~0.84).

## 3. Results

### 3.1. Spatial-Temporal Evolution Characteristics of INRPI

**Figure 3:**
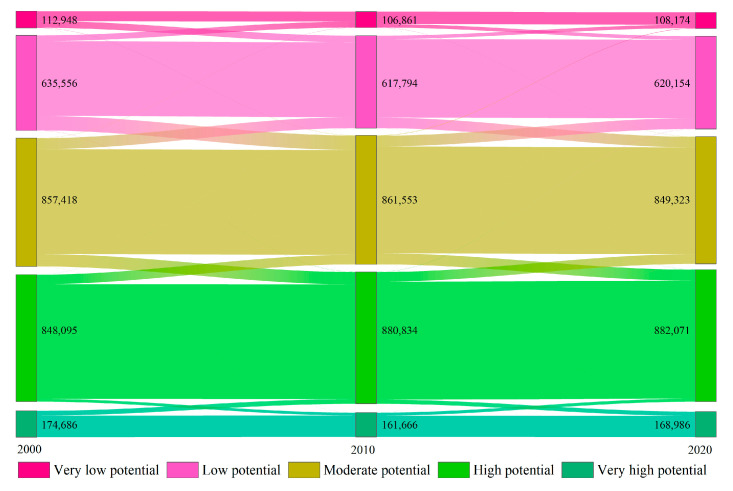
INRPI transition from 2000 to 2020.

The mean INRPI values for 2000, 2010, and 2020 were 0.4424, 0.4429, and 0.4433, respectively, showing a slightly increasing trend. Among the five INRPI levels, only areas with high potential showed an increase, with all others demonstrating a decreasing trend. The areas of the high potential level increased by 34,065 km^2^, while the areas with low potential decreased by 15,390 km^2^. To reflect the flow direction and rate for each INRPI level from 2000 to 2020 better, a transition matrix (Figure 3) was introduced in this study. From 2000 to 2010, areas of low potential transferred mainly to the moderate potential level (96,504 km^2^). Concurrently, areas with moderate potential transferred to the high potential level reaching 82,322 km^2^. From 2010 to 2020, areas of moderate potential transferred 73,531 km^2^ to the high potential level and 71,750 km^2^ to the low potential level. The areas of moderate potential increased by 70,296 km^2^ from the low potential level. Overall, the areas suitable for NBR in the QTP increased remarkably over the past 20 years.

**Figure 4:**
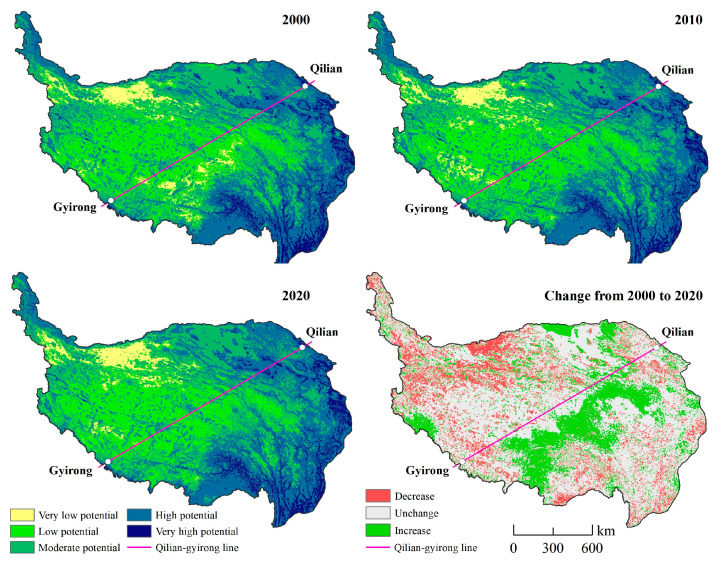
The spatial change in NBR from 2000 to 2020.

The distribution characteristics of the INRPI in the QTP from 2000 to 2020 are shown in Figure 4. In the last 20 years, the spatial pattern of NBR potential was relatively stable. The INRPI covering the QTP shows noticeable regional differences that gradually decline from the southeast to northwest. The very high and high potential areas are distributed mainly in the eastern and northwestern regions, especially the Hengduan Mountain region, Qilian Mountains, eastern Himalaya Mountains, and Pamir Plateau. The INRPI in these regions, with lakes and rivers, notably improved. Lakes and valleys are abundant, with diverse landscapes, sufficient oxygen, and suitable climate conditions. Areas of moderate potential existed in the northern regions, including the Qaidam Basin and Three-River-Source region. The Qaidam Basin is flat and provides more recreational opportunities and a lower plateau reaction risk index. The combination of rivers, grasslands, animals, and snowy mountains in the Three-River-Source region increases landscape diversity and attractiveness. However, both regions have poor recreation reception ability. The very low and low potential areas are concentrated primarily in the core area of the Qiangtang Plateau, which is the largest depopulated zone with a high plateau reaction, cold climate, and inconvenient transportation. Moreover, the very low potential areas transferred to the northwest. In general, from 2000 to 2020, the increased regions of INRPI were mainly located in the east of the Qilian-Gyirong line, the north of the Qaidam Basin, the Yarlung Zangbo River Basin, and the Sacred Mountains and Lakes (Kangrinboqe mountain, Mapam Yumco Lake), with a total area of 431,407 km^2^. However, the decreased areas of INRPI, which covered an area of 529,252 km^2^, were widespread in the QTP, especially in the northwest.

### 3.2. Spatial Differences along the Qilian-Gyirong Line

The mean INRPI values in the southeast of the Qilian-Gyirong line for 2000, 2010, and 2020 were 0.4759, 0.4792, and 0.4821, respectively. Values northwest of the line were 0.4086, 0.4063, and 0.4042, respectively. This indicates that the INRPI on both sides of the Qilian-Gyirong line maintained long-term stability with slight variations, increasing slightly in the southeast and decreasing slightly in the northwest.

**Figure 5:**
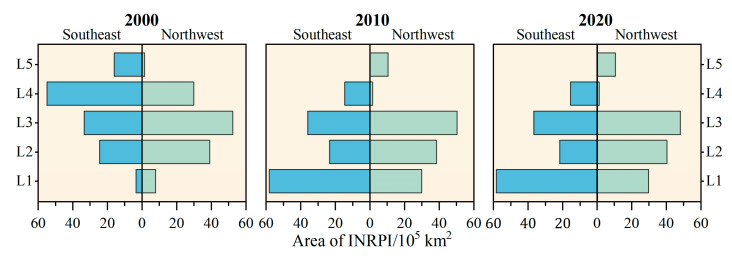
Difference in INRPI on both sides of the Qilian-Gyirong line (L1 is the very low potential; L2 is the low potential; L3 is the moderate potential; L4 is the high potential; and L5 is the very high potential).

As shown in Figure 4 and Figure 5, the region southeast of the Qilian-Gyirong line was dominated by high potential areas, followed by moderate potential areas, in total covering 66.82%, 71.17%, and 71.90% in 2000, 2010, and 2020, respectively. The area of moderate potential regions northwest of the Qilian-Gyirong line was the largest, followed by the low potential areas, whereas the very high potential regions had the smallest area. In the last 20 years, very low and low potential areas southeast of the Qilian-Gyirong line decreased distinctly by 33,681 km^2^ and 28,283 km^2^, respectively. Areas with moderate and high potential areas increased rapidly by a combined 67,230 km^2^. The opposite trend appeared in the region northwest of the Qilian-Gyirong line.

### 3.3. The Effects of Protected Areas on INRPI

The sensitive and fragile ecosystems in the QTP make it vulnerable to biodiversity loss and ecological destruction. Natural protected areas play a vital role in biodiversity conservation and regional ecological security [5,19]. Local governments have established various protected area categories (PAs) to protect biodiversity and ecosystems, including nature reserves, scenic areas, geological parks, forest parks, wetland parks, national parks, and biodiversity conservation priority areas. Considering the availability of boundaries, this research only analyzed the INRPI in the nature reserves, national parks, and biodiversity conservation priority areas. 

**Figure 6:**
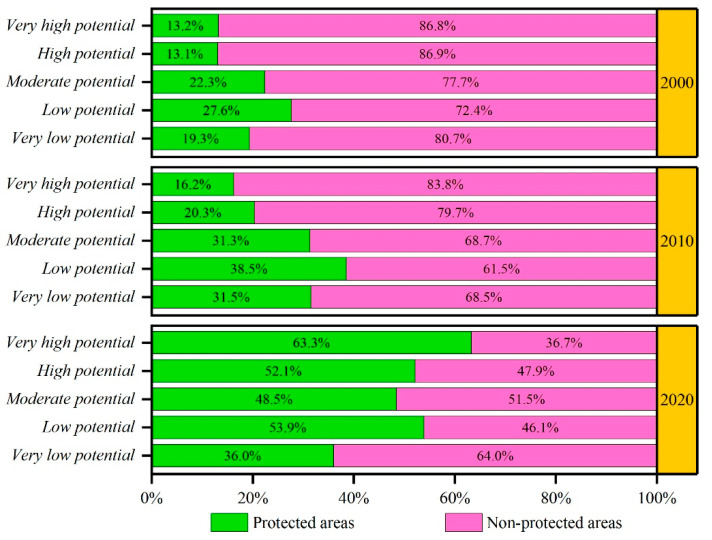
The proportion of INRPI in protected areas and non-protected areas from 2000 to 2020.

The results showed that the mean INRPI values in PAs were 0.4153, 0.4163, and 0.4490 in 2000, 2010, and 2020, respectively, demonstrating a continuously increasing trend over the last 20 years. Moreover, in 2020 the mean INRPI was higher within the PAs than in non-protected areas (NPAs). To understand the differences in the NBR potential between PAs and NPAs further, it is essential to compare the proportions and changes in different INRPI levels from 2000 to 2020 (Figure 6). There was an apparent tendency toward the growth of INRPI at various levels within PAs, particularly in 2020. Specifically, the results showed that, among all levels, the proportion of areas with very high potential demonstrated the highest growth rate (50.1%), followed by high potential (39%), low potential (26.3%), moderate potential (26.2%), and very low potential (16.7%). The areas of very high, high, and moderate potential in PAs increased by 83,993 km^2^, 348,847 km^2^, and 220,092 km^2^, respectively.

The Chinese government implemented China’s Biodiversity Conservation Strategy and Action Plan in 2011 and established national park system pilot projects (e.g., Three-River-Source, Giant Panda, Qilian Mountain, and Potasto) in the QTP since 2016. The implementation of these protection schemes and the construction of protected areas have protected many important biological and ecological resources, thereby improving landscape attractiveness. This implies that the protected areas played an important role in conserving and improving NBR potential. PAs are constructed for biodiversity conservation and ecological protection and provide various ecosystem services [30,45]. The NBR services provided by PAs should be adequately emphasized and rationally utilized.

### 3.4. Thresholds Identification: The Responses of INRPI to Elevation

**Figure 7:**
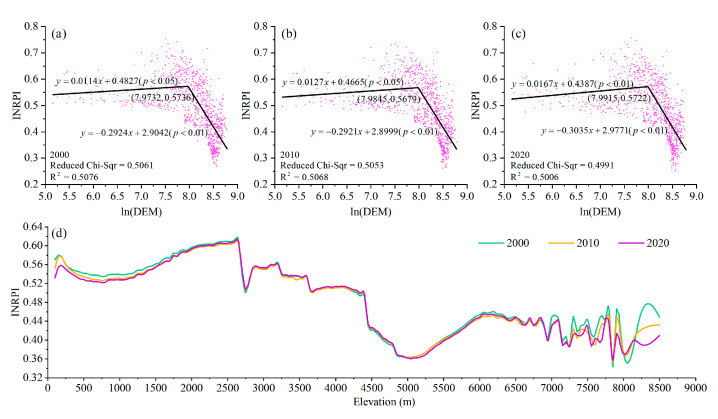
The relationship between elevation and INRPI from 2000 to 2020. (**a**–**c**) Correlations between DEM and INRPI in 2000, 2010, and 2020, respectively. (**d**) Distribution of INRPI in different elevation in 2000, 2010, and 2020, respectively.

We randomly selected 1000 points at different altitudes to explore the response of INRPI to elevation. The scatter diagrams comparing the INRPI and elevation from 2000 to 2020 are shown in Figure 7a–c. There were pronounced thresholds of the INRPI response to elevation that remained comparatively stable during the last 20 years. Before each threshold, there was a weak positive correlation between elevation and INRPI. However, INRPI decreased rapidly with elevation after the thresholds. For 2000, 2010, and 2020, when the elevation exceeded 2899, 2933, and 2952 m, respectively, the INRPI decreased dramatically with increasing elevation, and the slopes of the linear relationship were −0.2924, −0.2921, and −0.3035 (*p* < 0.001), respectively.

To understand the effects of elevation on the INRPI further, it is essential to compare the changes in the INRPI at different terrain gradients (Figure 7d). From 2000 to 2020, the relationships of INRPI with terrain gradient showed comparable trends. The INRPI increased from 0.52 to 0.62 at altitudes from 100 to 2650 m, and it showed a stepwise decline at altitudes exceeding 2650 m. The INRPI decreased rapidly at 2650–2750 m and increased slightly at altitudes ranging from 2750 to 2900 m. Overall, this indicates that 3000 m was a critical dividing line between elevations suitable and unsuitable for NBR in the QTP.

The complex topography of the QTP makes a remarkable difference in landscape, climate, biodiversity, and oxygen content. The vegetation types distributed in vertical zonation in the QTP cause heterogeneity in landscape patterns with elevation [31]. In general, landscape types in low-elevation areas are more diverse and abundant than those in high-elevation areas. This is also generally the case for the Hengduan Mountain region. The altitude of this region, which is a suitable climate comfort zone for human habitation and recreation, was relatively low [26]. The prevalence of high-altitude illnesses increases dramatically when people are exposed to altitudes exceeding 3000 m [27,39]. The population tends to live at 2600 to 2700 m [29], which determines the spatial pattern of recreation reception facilities. In conclusion, the differences caused by altitude, including landscape differences and climate differences, are the fundamental causes of the decline in recreational potential.

## 4. Discussion

### 4.1. The Advantages and Shortages of NBR Potential Index System

The suitability and importance of the selected evaluation indicators directly determine the reliability of the results. Consistent with previous studies [3,6,16,17], our research considers factors such as recreation resources, natural environment, and socioeconomic conditions as the basis for regional recreation development. In addition, the complex natural conditions of the QTP make it unique from other places. Therefore, some factors that affect recreation development should be taken into account, such as topographic relief, biodiversity, oxygen content, and plateau reaction. In particular, plateau reaction becomes a major obstacle limiting most tourists from low altitude areas to travel to the QTP [27]. In summary, our evaluation indicators reflect both the basic requirements of tourism potential and the special characteristics of the QTP.

However, we must also be aware of its shortcomings, namely that recreation reception ability is not fully considered. First, there is a lack of long-term socioeconomic statistical data in Tibet and Qinghai, especially at the township and county levels. However, the latest gridded socioeconomic statistics are not readily available. Although the application of nighttime light data for urbanization in the QTP attracted our interest and attention [46], it is notable that the population and economic activities of the Tibetan Plateau are mainly concentrated in cities.

### 4.2. Factors Influencing INRPI in the QTP

The spatial pattern of the INRPI in the QTP is related to the spatial distribution of ecological geographical elements. The complex topographic conditions in the QTP play a decisive role in the spatial distribution of the NBR potential. Our research revealed that INRPI gradually decreased with increasing elevation; similar results were observed by Wu [31]. The altitude of the QTP gradually increases from southeast to northwest, along with the harshness of the climate [26]. As the main restriction of NBR development [6], topographic relief has an evident influence on landscape attractiveness and recreational opportunities. Specifically, access to nature and recreational experience decreased with an increase in relief, especially in high-altitude areas. The pronounced spatial differences in landscape types lead to differences in recreational activities [20]. A clear contrast exists between the southeastern and northwestern areas of the QTP. Commonly, the suitability for NBR is improved in areas with dense vegetation because it provides abundant recreational resources, a comfortable climate, and adequate oxygen [3].

Meanwhile, the recreational potential in some regions of the QTP increased during the study period. As one of the most sensitive areas to global climate change, the QTP is undergoing notable warming and slightly increasing precipitation [28,47,48]. The tourism climate index has increased, and the climate comfort period has expanded [22]. Climate warming will gradually expand the distribution of vegetation, move the vegetation boundaries northwestward [49], and continue greening [50]. This may increase oxygen concentration, further lowering the health risks of hypoxia when conducting natural recreation activities [13]. Because of ecological restoration and conservation policies (e.g., Natural Forest Conservation Project, Grassland Ecological Protection and Construction Projects, Three North Shelterbelt Project, and Yangtze River Shelterbelt Project), the ecosystems in the QTP have undergone visible changes [14]. Correspondingly, the increasing wildlife populations and protected unique and fragile ecosystems on the plateau enhance landscape attractiveness and ensure sustainable utilization of natural recreation resources. Thus, strengthening the construction and management of PAs can maintain and improve the NBR potential, thereby promoting the realization and transformation of the ecological value of PAs. Furthermore, the length of highway in Qinghai and Tibet increased from 41,182 km in 2000 to 203,369 km in 2020. These infrastructures, such as trails and roads, provide accessibility for in-situ experiential interactions with nature [45].

In general, natural geographical conditions in the QTP determine the basic pattern of NBR, while climate change, ecological restoration and conservation policies, and the improvement of socioeconomic conditions play a key role in enhancing the potential for recreation.

### 4.3. Policy Implications

To promote the sustainable development of regional tourism effectively, the following measures deserve attention.

First, the regions for NBR are still concentrated in famous areas, such as the Shangri-La and Qinghai Lake. This research revealed immense potential for developing NBR in several regions, including the Qaidam Basin, Sacred Mountains and Lakes, Three-River-Source regions, and Yarlung Zangbo River Basin, which is consistent with the results of our field research. To cope with these gaps, we highlighted the comprehensive dynamic assessment framework and optimized NBR space, focusing on the impact of global climate change on NBR in the QTP and its initiative response. Furthermore, it is important to improve infrastructure to enhance recreation reception capacity.

Second, local governments have long overemphasized ecological protection. The efficiency of the development and utilization of natural recreation is limited, particularly in protected areas. In addition to the ecological values provided by protected areas, recreational and cultural values deserve more attention [30]. Recreationists can gain inspiration from nature, improve physical and psychological experiences, and support their identity by experiencing biodiversity, especially flagship and iconic species [19]. Thus, recreational zones in protected areas should account for the need for natural experience and education. Limiting access, especially in winter, is an effective management measure to strike a balance between ecological protection and recreational development [19].

## 5. Conclusions and Directions for Further Research

Addressing the challenge of increased demand for natural experiences and connectedness to nature after COVID-19 requires understanding the spatiotemporal variations of NBR, which supports NBR planning and management. This study established the integrated NBR potential model coupling the AHP-entropy method and GIS to evaluate the INRPI in the QTP, which combines both the merits of original data and experts’ experience. Using this model, the spatiotemporal characteristics of the NBR in 2000, 2010, and 2020 were analyzed. The following conclusions were obtained: (1) From 2000 to 2020, there was a stable spatial distribution pattern of INRPI in the QTP. The very high and high potential areas were scattered in the eastern and northwestern regions, while the very low and low potential areas were distributed in the core area of the Qiangtang Plateau. In the last 20 years, the low potential areas decreased by 15,390 km^2^. However, there was an increasing tendency in the high potential areas, expanding by 34,065 km^2^. (2) The regional differentiation characteristics of the INRPI can be characterized by the Qilian-Gyirong line. From 2000 to 2020, the mean values of the INRPI in the southeast were remarkably higher than that in the northwest. The southeast regions were dominated by high and moderate potential areas, which increased from 66.82% in 2000 to 71.90% in 2020. (3) Protected areas had higher INRPI scores than non-protected areas, showing an increasing trend with the proportion of different INRPI levels from 2000 to 2020. (4) There is a terrain gradient effect in the change in INRPI in the QTP. The INRPI declined rapidly when the elevation exceeded 3000 m, indicating a threshold for the effect of altitude on recreational potential. These results can provide governments and enterprises with information that can support recreational spatial planning and optimization and assist them in adopting mitigation and adaptation strategies for the sustainable management of natural recreation in plateau regions.

Despite its merits and contributions to the evaluation of spatial-temporal dynamics and policy implications of NBR in the QTP, there are limitations that should be addressed in the future. In addition to the AHP-entropy evaluation method, various methodological approaches have been applied to assess recreation potential, such as the fuzzy-analytic hierarchy process [16], Fuzzy DEMATEL MCDA model [17], and ordered weighted averaging model [3], while the potential and effectiveness of these methods remain to be discussed. The contribution of NBR to the number of recreationists and regional economic development needs further review, and can be predicted and simulated by the application of big data in the future [11]. Linking NBR with urban planning to build more livable future environments is an important issue for the future. In addition, NBR can cause some ecological problems [6,51]. Thus, the way NBR interacts with ecological conservation and ecosystem services needs to be refined, which should be a priority to be solved in the future to support sustainable tourism.

## Figures and Tables

**Table 2:** Weight of evaluation indicator of NBR in the QTP during 2000–2020.

Indicator	AHP	2000		2010		2020	
		EEM	$W_{i}$	EEM	$W_{i}$	EEM	$W_{i}$
SHDI	0.1577	0.2583	0.2749	0.2573	0.2776	0.2534	0.2765
RDLS	0.1577	0.0107	0.0559	0.0111	0.0575	0.0107	0.0568
HQ	0.0526	0.0786	0.0876	0.0764	0.0874	0.0738	0.0862
DTPA	0.1851	0.0155	0.0730	0.0111	0.0623	0.0107	0.0616
NDVI	0.0169	0.0544	0.0413	0.0613	0.0444	0.0592	0.0438
DTL	0.0629	0.0155	0.0426	0.0111	0.0363	0.0107	0.0359
DTR	0.0259	0.0107	0.0227	0.0111	0.0233	0.0107	0.0230
DTG	0.0771	0.0058	0.0289	0.0060	0.0297	0.0058	0.0294
OC	0.0693	0.0107	0.0371	0.0111	0.0381	0.0107	0.0377
PRRI	0.0563	0.3117	0.1804	0.3226	0.1858	0.3116	0.1832
TEM	0.0221	0.0107	0.0209	0.0111	0.0215	0.0058	0.0157
PRE	0.0135	0.1757	0.0663	0.1668	0.0654	0.1903	0.0701
TNI	0.0074	0.0155	0.0146	0.0161	0.0150	0.0155	0.0148
DTC	0.0159	0.0204	0.0245	0.0211	0.0252	0.0204	0.0249
TA	0.0796	0.0058	0.0293	0.0060	0.0302	0.0107	0.0404

## Data Availability

The data that support the findings of this study are openly obtained from the Zenodo platform (https://zenodo.org/, accessed on 20 February 2022), the National Tibetan Plateau Data Center (https://data.tpdc.ac.cn/, accessed on 20 February 2022), the Geospatial Data Cloud (http://www.gscloud.cn/, accessed on 20 February 2022), and the United States Geological Survey (https://www.usgs.gov/, accessed on 20 February 2022).
